# Genome-Wide Association Study Identifies a Plant-Height—Associated Gene *OsPG3* in a Population of Commercial Rice Varieties

**DOI:** 10.3390/ijms241411454

**Published:** 2023-07-14

**Authors:** Shasha Peng, Yanchen Liu, Yuchen Xu, Jianhua Zhao, Peng Gao, Qi Liu, Shuangyong Yan, Yinghui Xiao, Shi-Min Zuo, Houxiang Kang

**Affiliations:** 1 College of Agronomy, Hunan Agricultural University, Changsha 410128, China; pengshasha0914@gmail.com (S.P.); ippxuyuchen@163.com (Y.X.); 2 State Key Laboratory for Biology of Plant Diseases and Insect Pests, Institute of Plant Protection, Chinese Academy of Agricultural Sciences, Beijing 100193, China; liuyc@big.ac.cn (Y.L.); qiliu6786@gmail.com (Q.L.); 3 Jiangsu Key Laboratory of Crop Genomics and Molecular Breeding/Zhongshan Biological Breeding Laboratory/Key Laboratory of Plant Functional Genomics of the Ministry of Education, Agricultural College of Yangzhou University, Yangzhou 225009, China; dx120180075@stu.yzu.edu.cn (J.Z.); yzugaopeng@163.com (P.G.); smzuo@yzu.edu.cn (S.-M.Z.); 4 Tianjin Key Laboratory of Crop Genetic Breeding, Tianjin Crop Research Institute, Tianjin Academy of Agriculture Sciences, Tianjin 300112, China; bioponser@gmail.com

**Keywords:** rice plant height, genome-wide association study, polygalacturonase, CRISPR/Cas9 gene editing, cell wall

## Abstract

Plant height is one of the most crucial components of plant structure. However, due to its complexity, the genetic architecture of rice plant height has not been fully elucidated. In this study, we performed a genome-wide association study (GWAS) to determine rice plant height using 178 commercial rice varieties and identified 37 loci associated with rice plant height (LAPH). Among these loci, in LAPH2, we identified a polygalacturonase gene, *OsPG3*, which was genetically and functionally associated with rice plant height. The rice plant exhibits a super dwarf phenotype when the knockout of the *OsPG3* gene occurs via CRISPR-Cas9 gene-editing technology. RNA-Seq analysis indicated that *OsPG3* modulates the expression of genes involved in phytohormone metabolism and cell-wall-biosynthesis pathways. Our findings suggest that *OsPG3* plays a vital role in controlling rice plant height by regulating cell wall biosynthesis. Given that rice architecture is one of the most critical phenotypes in rice breeding, *OsPG3* has potential in rice’s molecular design breeding toward an ideal plant height.

## 1. Introduction

Rice (*Oryza sativa* L.) is a crucial crop that serves as a staple food for more than half of the world’s population. The increasing global population, which is expected to reach 8.5 billion by 2030 and 9.7 billion by 2050, poses a significant challenge to food production [1]. Additionally, agricultural land degradation, farmland reduction, and environmental contamination further exacerbate the issue of food production [2,3]. Therefore, improving rice yields has been a major focus of agricultural research.

Plant height (PH) is one of the most important agronomic traits of rice. The introduction of semi-dwarf varieties of rice and wheat during the ‘Green Revolution’ in the 1960s was a significant breakthrough for crop yields [4,5]. Semi-dwarfism, characterized by shorter PH and stronger stems, has been a valuable trait in rice breeding because it enhances yield and reduces lodging [6,7]. Thus, understanding the genetic mechanisms underlying rice PH is crucial for crop improvement.

Previous research has demonstrated that PH is primarily regulated by numerous hormones [4], including gibberellin (GA) [5,6,7,8,9], brassinosteroid (BR) [10,11,12], indole-3-acetic acid (Aux/IAA) [13,14,15], and strigolactones (SLs) [16,17]. GA plays a vital role in promoting stem and internode elongation. For instance, the deficiency of the GA receptor GIBBERELLIN INSENSITIVE DWARF1(GID1) resulted in a dwarf phenotype in rice [18]. BR, a class of poly-hydroxysteroid plant hormones, significantly impacts the growth and development of rice plants [19,20]. *OsBRI1*, which is homologous to the Arabidopsis *BRI1* gene, encodes a putative BR receptor, and the loss-of-function of OsBRI1 prevents internode elongation [21]. Aux/IAA, which predominates among plant auxins, is crucial for various plant growth and development activities, including cell division, differentiation, elongation, floral and vascular development, tropism, and embryogenesis. The overexpression of *OsIAA1* resulted in decreased auxin sensitivity and plant height [22].

Besides plant-hormones-related pathways, several other rice PH-associated genes were identified. These genes are involved in various processes, including the development of cell walls, cytosolic glutamine synthesis, RNA editing, cell division, and fatty acid metabolism [23,24,25,26,27]. Plant cell walls comprise polysaccharides, including cellulose and pectin, which are important for mechanical strength, flexibility, and wall permeability [28]. The cell-wall-associated receptor-like kinase OsWAK10 and its variants regulate cell wall signaling and control the magnitude of secondary wall cellulose synthesis, thereby controlling rice PH [29].

The genome-wide association study (GWAS) is an efficient method for identifying loci associated with specific traits [30]. Over the past two decades, GWAS has been widely used to identify quantitative trait loci (QTLs) and genes associated with rice’s numerous agronomic traits [31,32]. In this study, we conducted a GWAS to investigate the genetic basis of rice PH and identified dozens of PH-associated loci. Further haplotype analysis allowed us to focus on a candidate gene, *OsPG3,* which encodes polygalacturonase (PG), a pectin-degrading enzyme. The gene knockout mutant *ospg3* exhibits a decreased rice PH phenotype. We performed phylogenetic and structural analysis to predict its potential functions and detected its expression profile using multiple tissue samples throughout rice’s development stages. In addition, RNA-Seq analysis indicated that *OsPG3* modulates the expression of genes involved in phytohormones metabolism and cell wall biosynthesis pathways. In short, our results suggest that *OsPG3* is associated with rice PH, and the suitable *OsPG3* haplotypes, through further screening, will help the development of rice varieties with ideal rice PH.

## 2. Results

### 2.1. Variation in Plant Height in 178 Homozygous Commercial Rice Varieties

**Figure 1:**
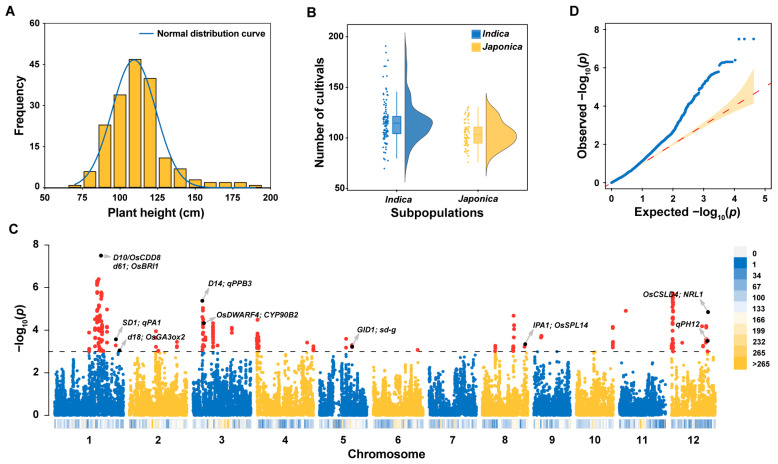
PH phenotypic distribution and genome-wide association scan in 178 commercial rice varieties. (**A**) Histogram illustrating PH diversity in the rice population. (**B**) PH differences between the two rice subpopulations, with each dot representing a rice variety. Boxplot lines indicate the average PH value for the two subpopulations. (**C**) Manhattan plot of the genome-wide *p*-values from rice PH GWAS. The x-axis shows the SNPs along each chromosome, while the y-axis represents -log10 (*p*-value) for the association. Red dots indicate SNPs with *p*-values < 1 × 10^−3^. Grey arrows indicate the PH-associated loci co-located with known QTLs or genes. (**D**) Quantile–quantile plot of rice PH GWAS.

To explore the genetic variation of rice PH, we collected a set of 178 varieties comprised of 109 *indica* and 69 *japonica* rice subpopulations (Table S1). Then, we planted these accessions in the field in Yangzhou (Southeast China) and measured rice PH at maturate stage (Table S1). The rice PH exhibited a normal distribution ranging from 69.67 cm to 190.67 cm, with an average of 111.74 ± 19.45 cm (Figure 1A, Table S2). Consistent with previous research suggesting that *indica* rice generally has a higher average PH than *japonica* rice [33], we also observed a significantly higher average PH in *indica* rice compared to *japonica* rice, and the highest cultivar was from the *indica* rice subpopulation (Figure 1B). These results indicate that similar to the rice diversity germplasm resources [32], there is also diversity in the rice PH phenotype in the selected commercial rice variety population.

### 2.2. Identification of the Locus Associated with Plant Height (LAPH) and PH Candidate Genes through Genome-Wide Association Study

To identify the loci associated with plant height (LAPHs) in rice, we performed GWAS of the PH phenotypes and the genotypes of 42,469 single-nucleotide polymorphisms (SNPs) with a minor allele frequency (MAF) at least 0.05% in 178 commercial rice varieties. We used the mixture linear model (MLM) for the analysis and identified 314 SNPs significantly associated with PH (Figure 1C and Table S3). Based on the average size of the rice linkage disequilibrium (LD) decay [34], these 314 SNPs could be further divided into 37 non-redundant LAPH regions and distributed on rice chromosomes 1, 2, 3, 4, 5, 8, 9, 10, and 12, respectively. Among these LAPH regions, ten co-localized with previously reported quantitative trait loci (QTLs) or genes linked to rice PH phenotype (Figure 1C and Table S4). For instance, LAPH9 co-localized with the GA biosynthesis gene, *SD1* [7], and LAPH29 co-localized with the semi-dominant QTL, *Ideal Plant Architecture 1* (*IPA1*) [35].

**Figure 2:**
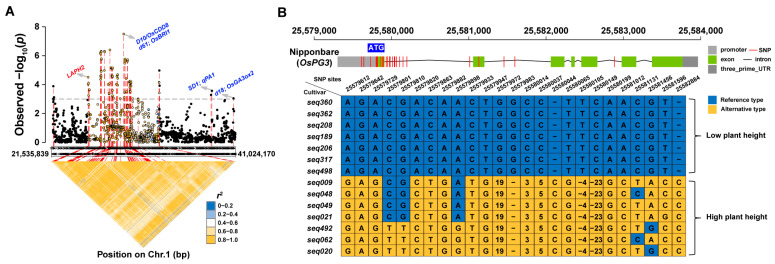
*OsPG3* is genetically linked to rice PH. (**A**) Genome-wide association signals and LD heatmap of *OsPG3* region on rice chromosome 1. The red-labeled loci are *OsPG3* locus associated with PH. Blue-labeled loci represent the three known QTLs or genes related to rice PH. (**B**) *OsPG3* sequence variation in different rice cultivars. The top part displays the *OsPG3* gene structure, while the bottom panel represents the detailed sequence variations in fourteen cultivars.

It is noteworthy that 27.03% (10 out of 37) of the LAPH regions identified in this study were on chromosome 1 (as shown in Figure 1C and Figure 2A and Table S4). Among those loci, we selected one previously unreported locus, LAPH2, for further investigation. The candidate genomic region spanned from 25,224 to 25,704 kb based on the LD block analysis around the top SNP (Figure 2A), which yielded 56 annotated genes (Table S5). Out of these genes, the number of genes annotated as retrotransposon proteins, expressed proteins, and hypothetical proteins were 6, 16, and 3, respectively. According to the type of protein encoded in this region, we focus on a gene, LOC_Os01g45060 (named *OsPG3*), that encodes a polygalacturonase (PG) for further study. The PG gene family is a large gene family in plants; it has been reported to be responsible for various cell-separation processes [36,37]. We then cloned and sequenced *OsPG3* in seven accessions with high PH and seven accessions with low PH (Figure 2B and Table S6). A total of 25 polymorphisms for *OsPG3* were identified across the 14 accessions (Figure 2B). Among them, 19 polymorphisms are distributed in the non-coding region, and 6 SNPs are located in the exon region of *OsPG3*. Among the six SNPs, three resulted in missense mutations. Specifically, two missense mutations occurred in the first exon, and one occurred in the second exon (Figure 2B).

### 2.3. Phylogenetic Analysis of PG Genes

**Figure 3:**
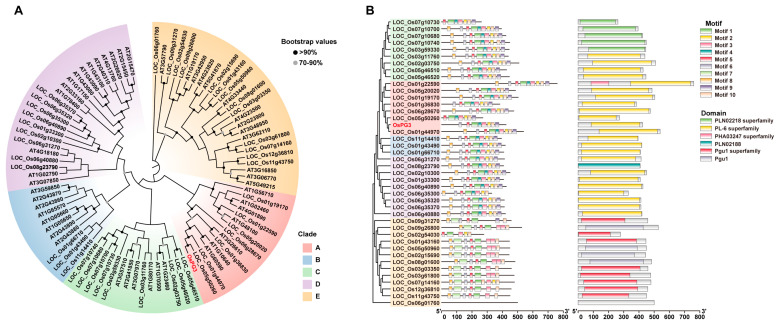
Phylogenetic analysis of PG Genes. (**A**) Phylogenetic analysis of PG gene families between rice and *Arabidopsis*. AT represents *Arabidopsis thaliana*; Os represents *Oryza sativa*. (**B**) MEME motif analysis identified domains of PG genes in rice.

Sequence alignment and conserved domains analysis were performed using the Basic Local Alignment Search Tool (BLAST) (https://blast.ncbi.nlm.nih.gov/Blast.cgi/, accessed on 23 March 2023) and Simple Modular Architecture Research Tool (SMART) [38] (https://smart.embl.de/, accessed on 24 March 2023). The analysis revealed that OsPG3 belongs to the pectin lyase-like superfamily and contains five or more parallel beta-helix repeat (PbH1) motifs that are conserved in this protein family (Figure S1). To investigate the relationships and gene structure of the PG genes, we utilized 44 PG genes [37,39] from rice and 49 PG genes [40] from *Arabidopsis* to construct a phylogenetic tree, which allowed us to classify these PG genes into 5 clades (Figure 3A). These five clades are consistent with the classification of *Arabidopsis* PGs, which were also divided into five clades (A to E) [37]. In the phylogenetic tree, *OsPG3* is in Clade A, where it is closely related to seven other rice PG genes (Figure 3A,B). It is noteworthy that one of these genes, LOC_Os01g19170 (hereafter named *OsPG1* or *PSL1*), has been shown to play a role in modifying rice cell wall structure. Loss-of-function mutation of this gene results in leaf rolling and leaf tip necrosis, as well as reduced plant height compared to wild-type plants [41,42]. These results suggest that *OsPG3* may also play a role in regulating the plant cell wall structure and influencing rice PH. To further analyze the conserved motifs of the rice PG genes, 44 rice PG protein sequences were aligned using the online tool MEME [43], and a total of 10 conserved motifs (Motif 1-10) were identified. Among these motifs, motifs 1, 2, 3, 4, 8, 9, and 10 represented the highly conserved domain PLN02218 and PL-6 of PG genes involved in clades A-D (as shown in Figure 3B). Motifs 1, 5, and 8 were present in all 44 PG protein sequences except for LOC_Os06g01760 (Figure 3B). Additionally, motif 7 was found exclusively in members of clade E (Figure 3B). Notably, *OsPG3* is classified in Clade A and contains all the PG protein motifs, except for motifs 7 and 8.

### 2.4. OsPG3 Is Essential for Maintaining Rice Plant Height

**Figure 4:**
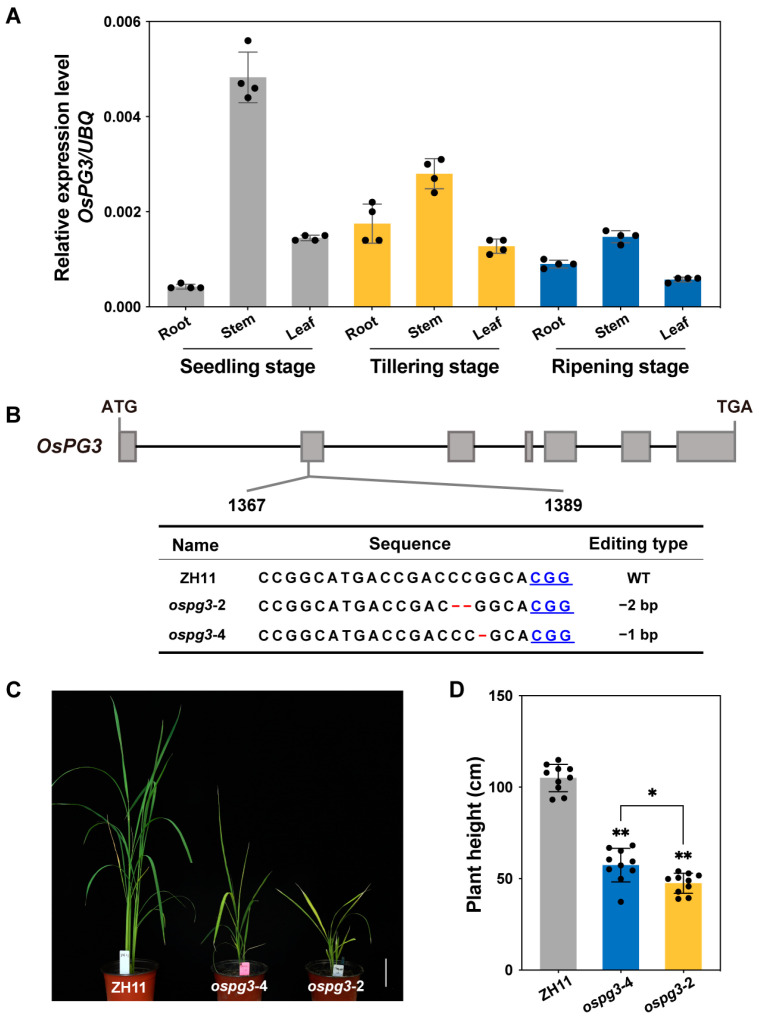
*OsPG3* gene knockout results in a dwarf phenotype in rice. (**A**) *OsPG3* transcription levels in various rice tissues. ‘Seedling’, ‘tillering’, and ‘ripening’ indicate different growth stages. (**B**) Editing types of *ospg3* mutant. The red-labeled ‘−−’ and ‘−’ indicate two base and one base deletions, respectively. (**C**) Phenotype comparison between wild-type ZH11 and two *ospg3* mutants. Scale bar = 10 cm. (**D**) Histogram of PH differences between wild-type ZH11 and two *ospg3* mutants. ‘**’ represents very significant differences (*p* < 0.01), and ‘*’ represents significant differences (*p* < 0.05).

To investigate the role of *OsPG3* in rice growth and development, we analyzed its spatial and temporal expression pattern. The total RNA was extracted from the roots, stems, and leaves of ZH11 at the seedling stage, tillering stage, and mature stage, respectively. RT-qPCR showed that, in three different growth stages, *OsPG3* transcripts were abundant in the stems rather than in the roots and leaves (Figure 4A). Gene-expression patterns are usually associated with their function [44], and the transcription levels of *OsPG3* in the stem were exceptionally higher than those in the root and leaf, indicating that *OsPG3* might play an important role in stem development.

Based on the results of the GWAS and expression analysis, it was hypothesized that *OsPG3* is involved in rice plant growth. To further validate the function of *OsPG3*, we generated a CRISPR/Cas9 knockout mutant *ospg3* using the *japonica* rice cultivar Zhonghua 11 (ZH11) through *Agrobacterium*-mediated transformation. Two independent homozygous lines, *ospg3*-2 and *ospg3*-4, were used for further investigation (as shown in Figure 4B). The gene-knockout mutant exhibited a plant height that was over 40% shorter than the corresponding wild-type plants at the reproductive stage (Figure 4C,D). In addition, we observed a significant decrease in plant height and shoot length in the *ospg3* mutant compared to the wild-type rice at the seedling stage, as shown in Figure S2. These results indicate that the *OsPG3* gene plays a crucial role in rice normal growth and development, as its knockout resulted in a super-dwarf phenotype.

### 2.5. OsPG3 Modulates the Expression of Genes Involved in Phytohormone Metabolism and Cell-Wall-Biosynthesis Pathways

**Figure 5:**
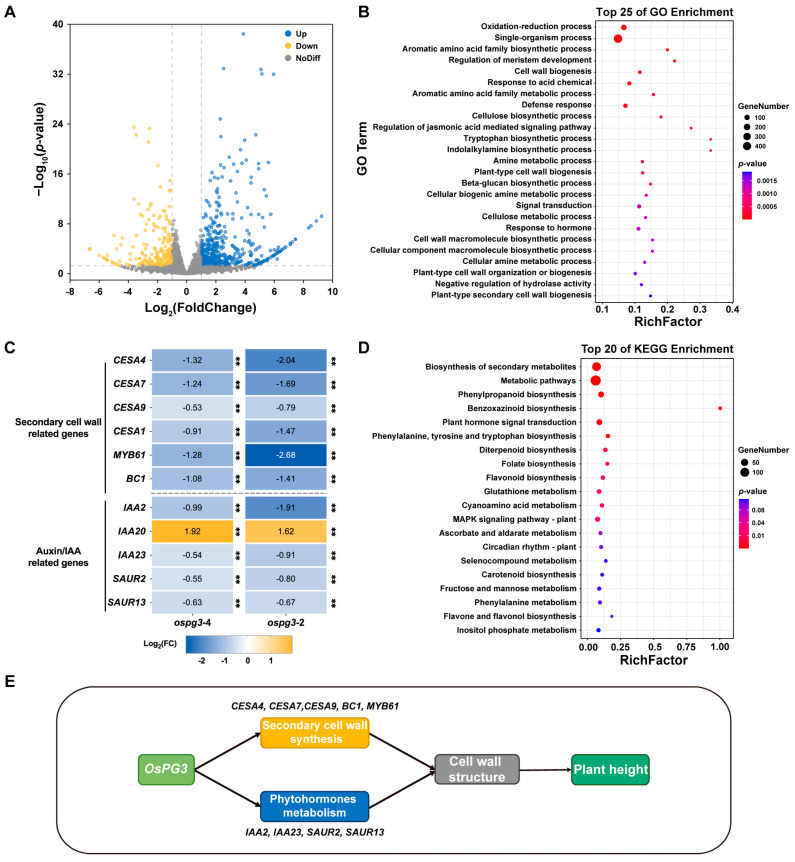
Differentially expressed genes in rice stem identified by RNA-Seq comparing wild-type ZH11 and *ospg3* mutant. (**A**) Volcano plot of differentially expressed genes. (**B**) Top 25 Gene Ontology (GO) enrichment categories. (**C**) Heat map showing the expression profiles of typical cell wall biosynthesis and regulatory genes and Auxin/IAA-related genes in *ospg3* knockout mutant. Yellow represents up-regulation, and blue represents down-regulation (** *p* < 0.01, *t*-test). (**D**) KEGG pathway enrichment of differentially expressed genes (DEGs). (**E**) The proposed model for *OsPG3* mediating plant height in rice.

In order to investigate the role of *OsPG3* in regulating rice PH, we performed a global transcriptional comparison between the knockout mutant *ospg3* and wild-type ZH11 plants using RNA-Seq analysis. A volcano plot was generated to visualize the differentially expressed genes (DEGs) (Figure 5A). There were 440 DEGs between ZH11 and the *ospg3* knockout lines, of which 266 DEGs were up-regulated and 174 DEGs were down-regulated. The GO enrichment analysis indicated that most of the differentially expressed genes (DEGs) were annotated to biological process terms (Figure 5B and Figure S3), with the most common term being the metabolic process, indicating that *OsPG3* has a wide-ranging effect on metabolic activities. GO analysis also revealed that the DEGs were involved in cell wall biogenesis, cellulose biosynthetic process, plant-type cell wall biogenesis, and plant-type cell wall organization or biogenesis (Figure 5B and Figure S3), indicating that *OsPG3* affects cell wall biosynthesis. In addition, the RNA-Seq data showed that the expression levels of genes related to development were apparently decreased (Figure S4). To validate our findings from RNA-Seq analysis, we conducted qRT-PCR analysis on the expression levels of the secondary cell wall and Auxin/IAA-related genes using the wild-type ZH11 and knockout mutant *ospg3*-4 and *ospg3*-2 plants; detailed results are presented in Figure 5C. Notably, the expressions of three cellulose synthase genes, CESA4 (LOC_Os01g54620), CESA7 (LOC_Os10g32980), and CESA9 (LOC_Os09g25490), which are part of a cellulose-synthesizing complex involved in the synthesis of the secondary cell wall [45], were down-regulated in the *ospg3* mutant (Figure 5C and Figure S4). Moreover, the expression of *Brittle Culm1* (LOC_ Os03g30250), which encodes a COBRA-like protein precursor that regulates the biosynthesis of secondary cell walls [46], was also down-regulated in the *ospg3* mutant (Figure 5C and Figure S4). We further analyzed the DEGs using the KEGG database to identify enriched pathways (Figure 5D). We found that the main enriched pathways were the biosynthesis of secondary metabolites, metabolic pathways, and phenylpropanoid biosynthesis, indicating that *OsPG3* might regulate rice plant architecture through these metabolic processes (Figure 5E).

## 3. Discussion

Plant height is not only a crucial factor of plant architecture but also an important agronomic trait that is directly linked to yield potential [47]. Over the past few decades, several genes involved in regulating PH have been identified, many of which are involved in plant hormone regulation [48,49]. Notably, some additional dwarfing genes were found to participate in other pathways, such as cell wall development, cytosolic glutamine synthesis, and cell division [24,26,50]. In this study, we observed significant variations in PH among a set of 178 commercial rice varieties at the maturate stage (Figure 1A). We found that *indica* rice has a higher average PH than *japonica* rice across rice subpopulations (Figure 1B). The large PH variations in this rice population provided an ideal resource for dissecting the genetic basis of rice PH.

GWAS is an efficient method for identifying genomic regions associated with a given agronomic trait, including rice PH [51,52,53]. In this study, we performed a GWAS using the PH phenotypes of 178 commercial rice varieties, and the genotype data consisted of 42,469 SNPs (Figure 1C,D). We identified 37 LAPHs (Figure 1C, Table S4), of which 10 co-localized with the previously reported QTLs related to rice PH [7,9,11,13,18,21,23,35,54,55]. Additionally, our GWAS analysis revealed that most of the significant SNPs associated with plant height (PH) are located on chromosomes 1, 3, and 12, while there are few significant SNPs on chromosomes 6, 7, and 11. These chromosomes contain genes previously reported to be related to PH, such as *OsMPH1* on chromosome 6, which, when overexpressed, increases PH by elongating internode cell length [56] and loss-of-function mutations in the rice homeobox gene *OSH15* on chromosome 7, which affect the internode architecture and result in dwarf plants [57]. However, we utilized a set of 178 commercial rice varieties in this study; the diversity of the commercial rice varieties may not be as comprehensive as that of natural populations. Finally, we identified a gene, *OsPG3* (LOC_Os01g45060), in LAPH2 on chromosome 1, with nucleotide polymorphisms linked to the rice PH phenotype (Figure 2A,B). *OsPG3* is annotated as a polygalacturonase (PG), a pectin-degrading enzyme involved in the cell wall modification process.

The cell wall not only provides mechanical strength to plant tissues but also regulates plant growth and development [58]. The function and regulation of plant cell wall hydrolytic enzymes have been well-studied [28]. These enzymes can alter the cell wall extensibility and cellular adhesion, leading to cell wall loosening, cell elongation, root tip sloughing, and fruit softening [39,58]. Plant PGs, among these enzymes, belong to the large Glycoside Hydrolase Family 28 (GH28), a member of the Glycoside Hydrolase (GH) superfamily in organisms [37,59]. PGs have been identified in various plants, including *Arabidopsis*, apples, and peaches [60,61,62]. In *Arabidopsis*, PGs encoded by *ADPG1* and *ADPG2* are essential for cell-separation and -expansion events during reproductive development [63]. Overexpression of *PGX1*, encoding a PG involved in cell expansion, led to enhanced hypocotyl elongation in etiolated *Arabidopsis* seedlings [64]. In rice, *PSL1* (*OsPG1*) has been shown to play a role in modifying the cell wall’s structure. Loss-of-function mutation of this gene results in leaf rolling and leaf tip necrosis, as well as reduced plant height compared to wild-type plants [41,42]. In our study, through phylogenetic and gene structure analyses, we identified *OsPG3* as a typical PG gene and classified it in Clade A of the PG gene family (Figure 3A,B and Figure S1).

The spatial–temporal expression profile showed that *OsPG3* was primarily expressed in the stems (Figure 4A). Loss-of-function of *OsPG3* resulted in a super dwarf phenotype (Figure 4C,D), indicating that *OsPG3* is essential for maintaining the height of rice plants. RNA-Seq analysis supported the *OsPG3* functions in the regulation of phytohormones metabolism and cell wall biosynthesis pathways (Figure 5). Thus, identifying and utilizing suitable natural alleles or creating appropriate alleles through gene editing are potential approaches for further utilizing *OsPG3* in rice molecular design breeding toward ideal plant height.

## 4. Materials and Methods

### 4.1. Plant Materials and Growth Conditions

A total of 178 homozygous commercial rice varieties were used for the plant height analysis, including 109 *indica* and 69 *japonica* varieties (as listed in Table S1). These varieties were cultivated using standard local practices during the natural growing season (April to September) of 2019 in Yangzhou, located in the Jiangsu province of China (32.39° N 119.42° E). Plant heights were measured after grain maturity; the data can be found in Tables S1 and S2.

### 4.2. Genome-Wide Association Study

GWAS analysis was performed using the mixture linear (MLM) method in TASSEL software (V5.2.87) [65]. A total of 42,469 SNPs with a minor allele frequency (MAF) > 0.05 were used for GWAS. The SNP data used in this study were personally obtained from a submitted paper titled ‘A natural variation of sheath blight-resistance receptor-like kinase 1 (SBRR1) improves sheath blight resistance in rice.’ It will be publicly available soon. The kinship matrix (K value) and the population structure Q value were used as random effects. The effective numbers of independent SNPs and significance thresholds were calculated following a previously described method [66]; the *p*-value threshold used in this study is 1E-03. Manhattan and Q-Q plots were generated using the CMplot *R* package [67]. Based on the average size of the LD decay blocks in rice [34], a 200 kb interval with at least two significantly associated SNPs was used for candidate gene analysis.

### 4.3. DNA Extraction and Gene Sequencing

Fourteen rice varieties, including seven with low plant height and seven with high plant height, were chosen for candidate gene sequencing. Rice seedling DNA was extracted using the CTAB method. The rice genome of the MSU7.0 version (http://rice.plantbiology.msu.edu/, accessed on 17 March 2022) was used as a reference for primer design (primers in Table S7). PCR and Sanger sequencing were then performed to obtain the sequences of candidate genes in all 14 rice varieties. The sequences were assembled and aligned using Snapgene6.0.2 and MEGA(V7.0) software [68].

### 4.4. Phylogenetic and Motif Analysis

Protein domain annotation was based on UniProt (https://www.uniprot.org, OsPG3 protein accession number: Q5VNN6_ORYSJ). A total of 44 rice PG protein sequences and 49 *Arabidopsis* PG protein sequences were obtained from blastp and tblastn searches against the National Center for Biotechnology Information (NCBI, https://www.ncbi.nlm.nih.gov/, accessed on 20 April 2023) database, using the glycosyl hydrolase family 28 (GH28, Pfam accession: PF00295) as the query. The phylogenetic tree was constructed using the neighbor-joining method with 1000 bootstrap pseudoreplicates in MEGA7. The resulting circle tree was visualized using the online tool tvBOT [69]. Conserved motifs in rice PGs protein sequences were identified using the MEME (v5.52) [43] (https://meme-suite.org/meme/tools/meme, accessed on 4 April 2023), with the following parameters: Classic mode, Zero or One Occurance Per Sequence (zoops); the number of motifs expected to be found was set to 25; and the motif width was set from 6 to 100 in ‘Advanced options.’ The conserved domains analysis was performed using the NLM’s Conserved Domains Database (CDD) (https://www.ncbi.nlm.nih.gov/Structure/bwrpsb/bwrpsb.cgi/, accessed on 20 April 2023). The MEME and conserved domain results were visualized using the TBtools software (V1.131) [70].

### 4.5. Vector Construction and Rice Transformation

The *ospg3* mutant was generated using CRISPR/Cas9 gene-editing technology in the ZH11 background to obtain a loss-of-function mutant. The second exon of *OsPG3* was targeted using sgRNA (5’-CCGGCATGACCGACCCGGCA-3’) designed using the online toolkit for CRISPR-based genome editing (CRISPR-GE) [71] (http://skl.scau.edu.cn/betarget/, accessed on 20 January 2019). The *Agrobacterium*-mediated transformation of rice callus (ZH11) and regeneration of rice plants for *OsPG3* gene editing was conducted following previously published protocols [72]. Homozygous *ospg3* mutants were screened via PCR and Sanger sequencing of the sgRNA target site (Tsingke Biotech, Beijing, China). Primer sequences used for plasmid construction and mutant identification are listed in Table S7.

### 4.6. Gene Expression Analysis

Spatial and temporal expression profiles of *OsPG3* were studied by collecting stem, leaf, and root tissues from ZH11 at the seedling, tillering, and ripening stages, respectively. RNA extraction and cDNA synthesis were performed using UNlQ-10 Column Trizol Total RNA Isolation Kit from Sangon Biotech (code: B511321, Shanghai, China) and HiScript II 1st Strand cDNA Synthesis Kit from Vazyme (code: R212-01, Nanjing, China), following the manufacturer’s instructions. For qRT-PCR, ChamQ SYBR qPCR Master Mix from Vazyme (code: Q311-02, Nanjing, China) was used with gene-specific primers (Table S7) and the following PCR cycling conditions: 95 °C for 30 s for pre-incubation, followed by 40 cycles of 95 °C for 5 s and 60 °C for 30 s for 2-step amplification. The rice *ubiquitin* (*UBQ*, LOC_Os03g13170) was used as an internal control for normalization. Transcript levels relative to *UBQ* were calculated using 2^−ΔΔCt^ methods, three biological replicates, and three technical replicates per sample.

### 4.7. RNA-Seq and Sequence Analysis

Rice plants were cultivated in pots within a greenhouse environment, and developing stem samples were collected from two-month-old wild-type (ZH11) and *ospg3* knockout mutant plants. Three biological replicates were collected for each sample at each time point. RNA extraction, library construction, and sequencing were carried out following the methods described in a previous study [73]. The clean RNA sequences were aligned to the rice cDNA file (MSUv7.0) using BWA software (V0.7.17), and the aligned data were viewed and sorted using Samtools. The number of reads aligned to each rice gene was calculated using the ‘grep’ and ‘awk’ commands in the Linux system. The *R* package DESeq2 was used to identify the differentially expressed genes (DEGs) [74]. The DEGs were determined using a |log_2_(FoldChange, FC)| ≥ 1 and a *p*-value < 0.05 as the criteria. The DEGs were classified via Gene Ontogeny (GO) annotation (http://www.geneontology.org/, accessed on 12 March 2023). The GO analyses were performed using AgriGO V2.0. The protein pathways were annotated using the Kyoto Encyclopedia of Genes and Genomes (KEGG) database. The online service tool KAAS was used to annotate the KEGG database description (http://www.genome.jp/tools/kaas/, accessed on 13 March 2023), and the resulting annotations were mapped onto the KEGG pathway database using KEGG MAPPER.

### 4.8. Data Analysis and Figures

The *p*-values were computed using Student’s t-test, ANOVA, two-tailed Mann–Whitney U-test, and an unpaired two-sample *t*-test, Pearson correlation (*r*^2^) was computed with GraphPad Prism (version 9.4.1), and *p* < 0.05 was considered a significant difference. The histograms were visualized in GraphPad Prism. The CloudRain plot and RegionalPlot were plotted using *R* package ggplot2 [75].

## Data Availability

The data presented in this study are available on request from the corresponding authors.

## Supplementary Materials

The following supporting information can be downloaded at: https://www.mdpi.com/article/10.3390/ijms241411454/s1.
