# Supplementary figures and images for: Genome-Wide Association Study Identifies a Plant-Height—Associated Gene OsPG3 in a Population of Commercial Rice Varieties
Source: Int J Mol Sci. 2023 Jul 14;24(14):11454. doi: 10.3390/ijms241411454 (PMC10380248; doi:10.3390/ijms241411454)

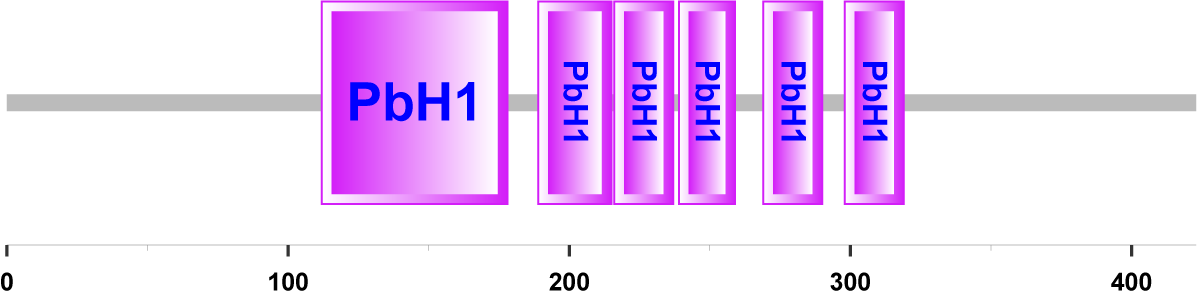

Supplement: Supplementary file 1 [file ijms-24-11454-s001.zip › ijms-2480811-supplementary/Figure S1.tif]

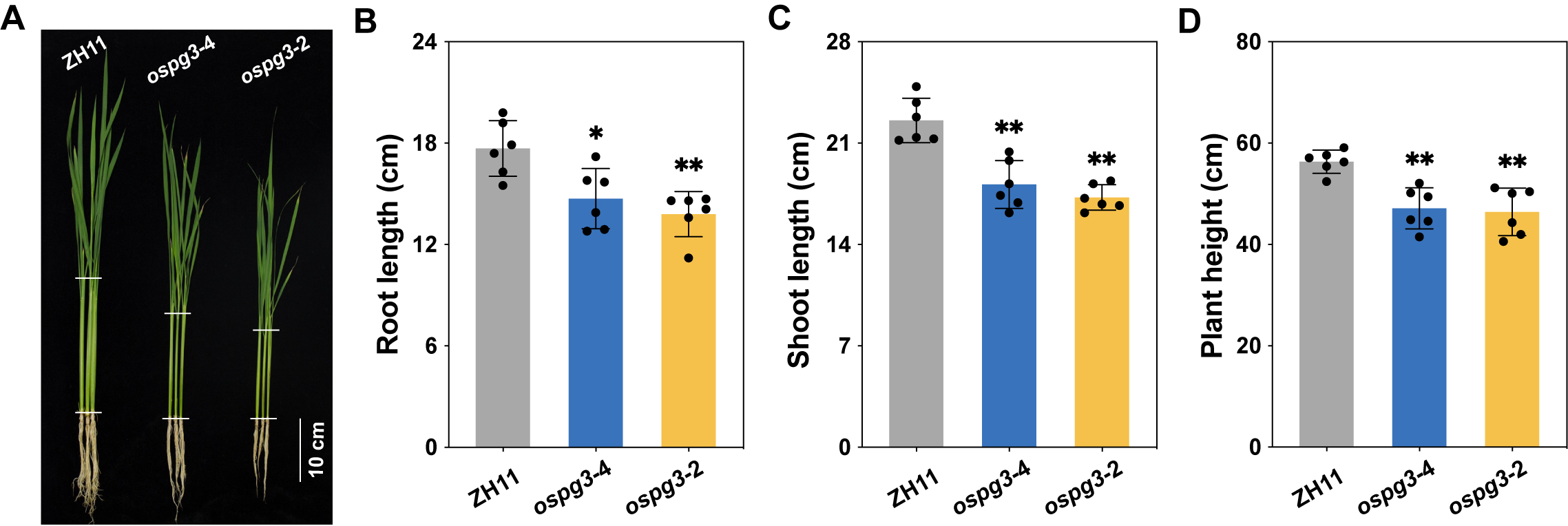

Supplement: Supplementary file 1 [file ijms-24-11454-s001.zip › ijms-2480811-supplementary/Figure S2.tif]

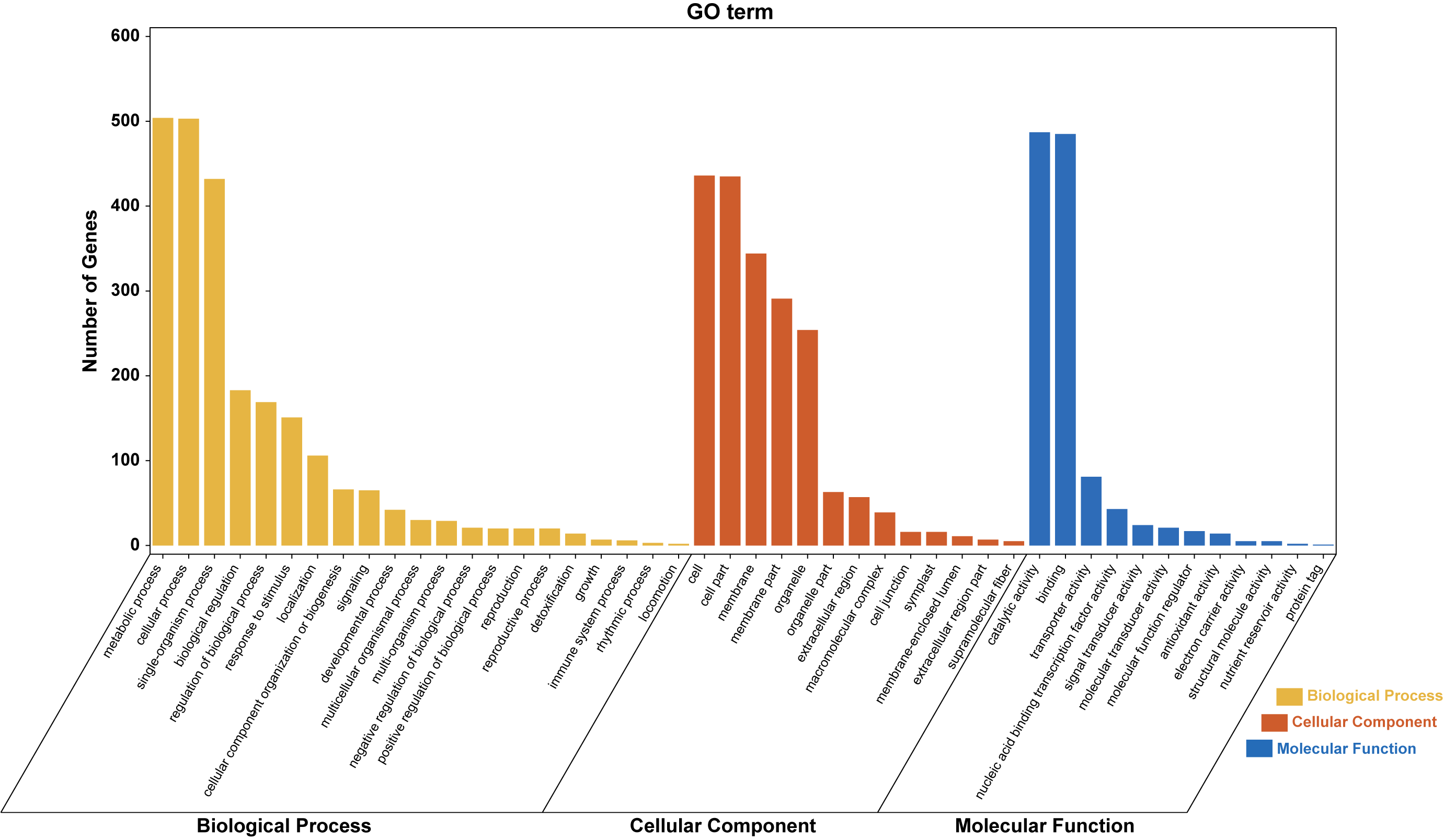

Supplement: Supplementary file 1 [file ijms-24-11454-s001.zip › ijms-2480811-supplementary/Figure S3.tif]

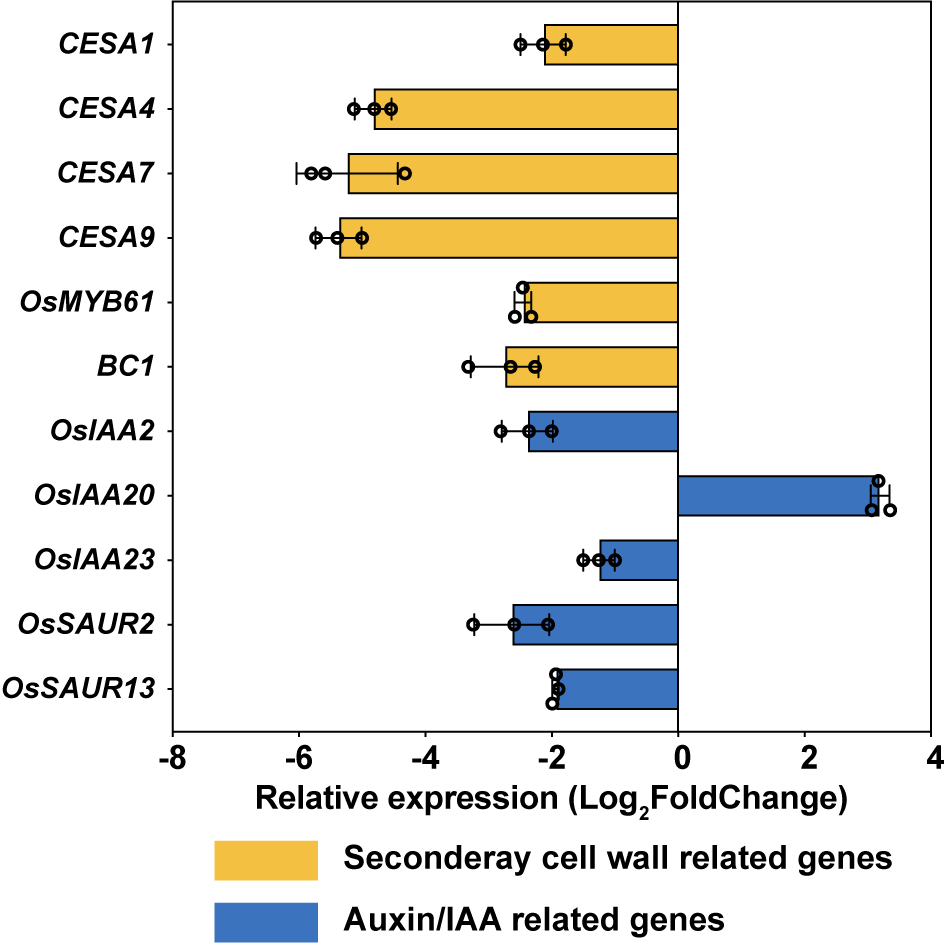

Supplement: Supplementary file 1 [file ijms-24-11454-s001.zip › ijms-2480811-supplementary/Figure S4.tif]
